# Antidepressant metabolite concentrations and metabolite-to-parent drug ratios in postmortem femoral blood

**DOI:** 10.1093/jat/bkag012

**Published:** 2026-02-15

**Authors:** Pirkko Kriikku, Ilkka Ojanperä

**Affiliations:** Forensic Toxicology Unit, Finnish Institute for Health and Welfare (THL), Helsinki, FI-00271, Finland; Department of Forensic Medicine, University of Helsinki, Helsinki, FI-00014, Finland; Forensic Toxicology Unit, Finnish Institute for Health and Welfare (THL), Helsinki, FI-00271, Finland; Department of Forensic Medicine, University of Helsinki, Helsinki, FI-00014, Finland

## Abstract

Antidepressants are commonly used psychiatric medications, many of them associated with significant toxicity in overdose. While established reference values are used in therapeutic monitoring of these drugs, there are less abundant reference data available in postmortem (PM) toxicology, especially concerning antidepressant metabolites. In this study, all such PM cases in 2016–2023 were retrieved from the Finnish national PM toxicology database in which an antidepressant drug and its metabolite were quantified concurrently in femoral venous blood, representing all causes of death (*N* = 3472). The results comprise the median concentrations of 11 antidepressants and their main metabolites, the parent drug plus metabolite sum concentrations, and the respective metabolite-to-parent drug ratio (MPR) values. The study revealed that the concentrations of antidepressants and their metabolites in PM blood generally increase in the following order according to the group studied: other cause of death than poisoning < implicated in fatal poisoning < principal finding in fatal antidepressant poisoning < single finding in fatal antidepressant poisoning. MPR was found to be a useful addition to concentration values in distinguishing acute poisoning death from other causes of death, showing lower MPR values in poisoning, and the results obtained in this study compared favorably with data previously published in the literature. The parent drug plus metabolite sum concentration did not provide significant additional information to parent drug only in PM diagnostics of poisoning.

## Introduction

Published drug reference concentrations generated from medicolegal casework have always been an important guide for assessing the role of poisoning as a cause of death. Gradually, increasingly more statistical data have accumulated on drug concentrations associated with therapeutic, toxic, or lethal levels [1–4]. For some drugs, concentrations in postmortem (PM) blood are close to antemortem (AM) plasma concentrations, while for many drugs PM increases or decreases occur mainly due to PM redistribution (PMR) [5]. An example of a common drug with similar AM and PM concentrations is the opioid oxycodone, whereas the common cardiac drug amlodipine and the common benzodiazepine lorazepam are examples with generally higher and lower PM concentration levels, respectively [6].

Antidepressants are a group of drugs with a high potential to cause fatal poisonings due to suicidal thoughts often being associated with depression and many antidepressants being toxic in overdose. Tricyclic antidepressants (TCAs) have in several studies demonstrated significantly higher fatal toxicity than selective serotonin reuptake inhibitors (SSRIs) or noradrenaline reuptake inhibitors (SNRIs) [7–11]. Most individual TCAs appear to show broadly similar toxicity [12].

Apart from case studies, few reports of fatal concentrations based on a large number of cases have been published in the literature. Our recent study provided summary statistics for fatal concentrations of 17 antidepressants in PM femoral blood and compared the results obtained with those of a Swedish research group [13]. As a rule, our results were consistent with the Swedish results, but there were also discrepancies, obviously due to different research settings. Normal and fatal PM concentration ranges overlapped in part in both datasets, making it sometimes difficult to interpret individual borderline cases using these reference ­concentrations.

Metabolites may provide an additional tool for diagnosis. In therapeutic drug monitoring (TDM), the sum of parent drug and active metabolite concentrations, i.e. the active moiety, is relevant for TDM-guided dosing but has rarely been utilized in PM toxicology. From a pharmacokinetic standpoint, determination of even non-active metabolites can be informative [14]. The metabolite-to-parent drug concentration ratios (MPR) measured in blood or urine have occasionally been used as an aid to ­interpretation in forensic toxicology, and for some substances, calculating MPR is part of daily casework [15]. However, few papers have reported reference metabolite and MPR data for antidepressants, although, already in 1989, Apple [16] recommended calculating MPR in liver and blood for assessment of cause of death from TCAs. In 2007, Reis *et al*. [17] in Sweden reported MPR and concentration data for 15 antidepressants in PM femoral blood in fatal poisonings and in control cases, and their article has long been the established source of this type of reference information.

In this study, our objective was to complement the previous research on fatal levels of antidepressants by providing metabolite concentration and MPR data on those drugs for which enough metabolite concentration results have accumulated in our database to date. These data from PM femoral venous blood are provided on 11 antidepressants and 13 metabolites in cases representing fatal poisonings as compared with other causes of death.

## Materials and methods

The medicolegal investigation process in Finland has been described earlier [13]. As part of a comprehensive PM toxicology panel [18], antidepressants were screened in urine by ultra-high-performance liquid chromatography coupled with high-resolution time-of-flight mass spectrometry [19]. Quantification in blood was performed by ultra-performance liquid chromatography coupled with photodiode array detection and charged aerosol detection (UPLC–PDA–CAD) [20], and with low-dose compounds, by liquid chromatography triple quadrupole mass spectrometry (LC–QqQ) using selected reaction monitoring. The quantification technique and the lower limit of quantification (LLOQ) in PM blood for each of the studied substances are given in Supplementary Table 1 (see online supplementary material).

In addition to the cause and manner of death, the forensic pathologist recorded on the death certificate all substances implicated in the poisoning and defined one (and only one) principal drug finding of poisoning to be used in poisoning statistics. The principal finding is the most important substance among those implicated in terms of toxicity. PM toxicology results and information from the death certificate were then entered into the national PM toxicology database maintained by the Finnish Institute for Health and Welfare (THL).

For this study, all such PM cases between 2016 and 2023 were retrieved from the Finnish national PM toxicology database in which at least one antidepressant drug metabolite was quantified along with the parent antidepressant drug in femoral venous blood. These cases included all causes of death.

The poisoning cases were divided into three groups according to the underlying cause of death, with the particular antidepressant being either implicated in the cause of death (Group 1), the principal finding of the cause of death (Group 2), or the only finding of the cause of death (Group 3), and each of these groups was statistically compared with Group 4, which comprises all other causes of death, including poisonings with other substances. Group 1 includes also Group 2 and 3 cases, and Group 2 also ­includes Group 3 cases.

Percentiles of drug and metabolite concentrations and MPRs were used to characterize the data. Statistical calculations were performed whenever there were at least 10 findings of a drug or its metabolite, using the Mann–Whitney *U* test for independent samples on IBM SPSS software (version 29). The Bonferroni correction was applied for multiple comparisons. A *P_(adj)_*-value <.05 was regarded as statistically significant.

The study was carried out based on the research permit THL/4850/6.02.00/2023, issued by THL, Finland.

## Results

The study material consisted of 3472 antidepressant-related death cases in which both parent drug and metabolite concentrations in PM femoral blood were available from the Finnish national PM toxicology database.

Table 1 shows the median PM concentrations of 11 antidepressants and their metabolites, the parent drug plus metabolite sum concentrations, and the respective MPRs.

**Table 1:** Concentrations (mg/L) of antidepressants and their metabolites, parent drug plus metabolite sum concentrations, and metabolite-to-parent drug concentration ratios.

		Group 1: Implicated in fatal poisoning			Group 2: Principal finding in fatal poisoning			Group 3: Single finding in fatal poisoning			Group 4: Other cause of death	
	*N* total	*N*	Median	*P*-value _(adj)_	*N*	Median	*P*-value _(adj)_	*N*	Median	*P*-value _(adj)_	*N*	Median
**Amitriptyline**	518	171	1.7	<.003	108	2.5	<.003	53	3.2	<.003	347	0.39
Nortriptyline			0.49	<.003		0.58	<.003		0.75	<.003		0.19
Amitriptyline + nortriptyline			2.2	<.003		3.4	<.003		4.2	<.003		0.61
Nortriptyline/amitriptyline			0.31	<.003		0.27	<.003		0.25	<.003		0.58
**Bupropion (with hydroxybupropion)**	212	47	1.1	<.003	27	2.3	<.003	10	3.3	<.003	165	0.12
Hydroxybupropion			1.8	<.003		2.0	<.003		2.9	<.003		0.26
Bupropion + hydroxybupropion			3.7	<.003		4.9	<.003		6.8	<.003		0.43
Hydroxybupropion/bupropion			0.71	<.003		0.59	.015		0.57	.030		2.0
**Bupropion (with threohydrobupropion)**	212	45	1.1	<.003	26	2.3	<.003	10	3.3	<.003	167	0.12
Threohydrobupropion			4.0	<.003		4.2	<.003		11	<.003		1.2
Bupropion + threohydrobupropion			5.3	<.003		7.7	<.003		14	<.003		1.4
Threohydrobupropion/bupropion			3.0	<.003		2.5	.015		2.8	.030		9.6
**Bupropion + hydroxybupropion + threohydrobupropion**	209	45	8.0	<.003	26	8.6	<.003	10	17	<.003	164	1.9
**Citalopram**	1316	52	1.6	<.003	16	3.2	<.003	3	13		1264	0.37
Norcitalopram			0.31	<.003		0.61	<.003		0.56			0.12
Citalopram + norcitalopram			2.2	<.003		3.8	<.003		14			0.51
Norcitalopram/citalopram			0.20	<.003		0.12	<.003		0.11			0.36
**Clomipramine**	33	10	1.5	<.003	8	1.9		3	2.5		23	0.40
Norclomipramine			2.3	.027		3.2			2.9			0.62
Clomipramine + norclomipramine			3.9	.027		4.6			5.1			0.93
Norclomipramine/clomipramine			1.4	1		1.4			0.91			1.8
**Doxepin**	74	25	2.8	<.003	18	2.7	<.003	6	4.2		49	0.44
Nordoxepin			0.90	<.003		0.78	<.003		2.1			0.20
Doxepin + nordoxepin			4.1	<.003		4.2	<.003		6.3			0.64
Nordoxepin/doxepin			0.29	.099		0.26	.078		0.31			0.53
**Fluoxetine**	117	11	2.5	<.003	3	2.4		0			106	0.49
Norfluoxetine			0.98	.024		0.96						0.55
Fluoxetine + norfluoxetine			3.3	<.003		3.3						1.0
Norfluoxetine/fluoxetine			0.36	.006		0.36						1.1
**Mianserin**	19	3	0.35		1	1.6		0			16	0.17
Normianserin			0.15			0.15						0.18
Mianserin + normianserin			0.87			1.8						0.39
Normianserin/mianserin			0.39			0.09						0.79
**Mirtazapine**	107	11	1.6	<.003	4	1.8		2	17		96	0.29
Normirtazapine			0.47	<.003		0.35			0.54			0.13
Mirtazapine + normirtazapine			2.0	<.003		2.2			18			0.44
Normirtazapine/mirtazapine			0.24	.036		0.17			0.42			0.44
**Sertraline**	238	16	1.3	<.003	10	1.7	<.003	8	1.9		222	0.25
Norsertraline			1.8	<.003		2.8	<.003		3.1			0.54
Sertraline + norsertraline			3.4	<.003		3.8	<.003		4.5			0.83
Norsertraline/sertraline			1.7	.051		2.2	1		2.7			2.3
**Trimipramine**	15	5	2.5		4	2.6		2	2.6		10	0.88
Nortrimipramine			0.78			0.65			0.72			0.87
Trimipramine + nortrimipramine			4.1			3.1			3.3			2.1
Nortrimipramine/trimipramine			0.36			0.28			0.28			0.52
**Venlafaxine (with *O*-desmethylvenlafaxine)**	793	117	2.6	<.003	62	6.6	<.003	20	9.1	<.003	676	0.55
*O*-desmethylvenlafaxine			1.1	<.003		2.2	<.003		3.5	<.003		0.52
Venlafaxine + *O*-desmethylvenlafaxine			3.6	<.003		10	<.003		13	<.003		1.2
*O*-desmethylvenlafaxine/venlafaxine			0.35	<.003		0.26	<.003		0.28	<.003		1.1
**Venlafaxine (with norvenlafaxine)**	204	51	3.0	<.003	33	4.3	<.003	8	12		153	1.0
Norvenlafaxine			0.59	<.003		0.98	<.003		1.7			0.22
Venlafaxine + norvenlafaxine			3.7	<.003		5.8	<.003		14			1.2
Norvenlafaxine/venlafaxine			0.15	.021		0.14	.036		0.16			0.24
**Venlafaxine + norvenlafaxine + *O*-desmethylvenlafaxine**	177	45	4.3	<.003	27	6.7	<.003	8	19		132	2.1

Groups 1, 2, and 3 are each compared separately with Group 4 for statistical significance (*P*-value_(adj)_), if *N* ≥ 10.

A general trend is observed that, provided there are ≥10 cases in each group, the median concentrations and median sum concentrations of antidepressants and metabolites are higher in poisoning cases (Groups 1–3) than in other causes of death (Group 4), and the concentrations in poisoning cases increase in the order Group 1 < Group 2 < Group 3. A difference is also almost invariably observed in the median MPR between poisoning cases and other causes of death such that the MPR value is lower in poisonings.

Using the same grouping, lower (10th), median, and upper (90th) percentiles for the concentrations and MPRs are presented in Supplementary Table 2 (see online supplementary material). The percentile distribution shows that despite significant differences between the medians there is considerable overlap ­between the groups.

Six case examples are provided in Supplementary Table 3 (see online supplementary material) to illustrate the use of reference drug concentrations and MPR values for interpretation.

## Discussion

Our study revealed that the concentrations of antidepressants and their metabolites in PM blood generally increase in the following order: Group 4 (other cause of death than antidepressant-related poisoning) < Group 1 (implicated in fatal antidepressant-related poisoning) < Group 2 (principal finding in fatal antidepressant poisoning) < Group 3 (single finding in fatal antidepressant poisoning).

However, when comparing the parent drug concentration with the parent drug plus metabolite sum concentration for each antidepressant, the sum concentrations do not provide significant additional information in PM diagnostics of poisonings. This result can be reached by examining the ratio of the median concentration of each poisoning group (Groups 1–3) to the median concentration of other causes of death (Group 4). Yet, since sum concentrations are commonly used in the TDM of many antidepressants in clinical pharmacology, the reference values for the corresponding PM sum concentrations may occasionally be relevant in PM toxicology. Furthermore, since PM total concentrations have seldom been published before [21], the data provided here is new.

**Figure 1:**
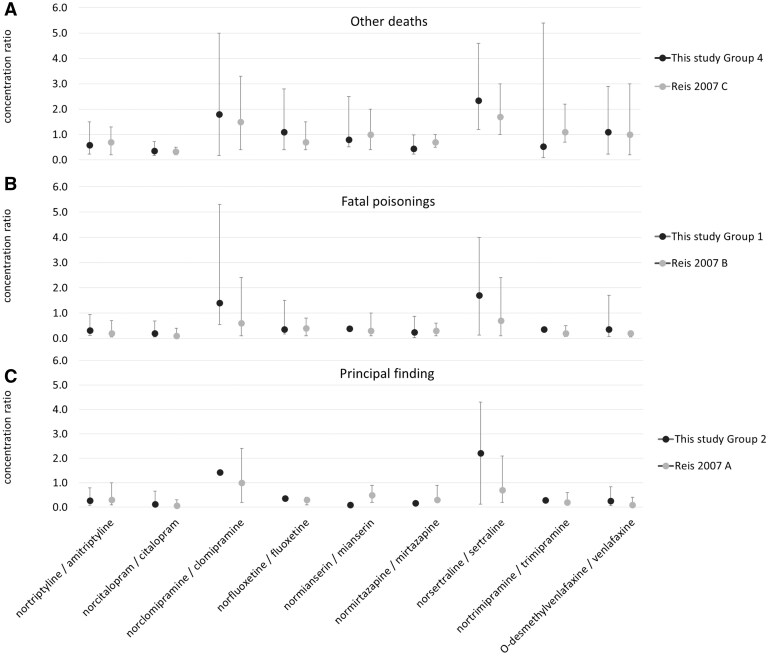
Comparison of metabolite-to-parent drug concentration ratios in various death groups between this study and that of Reis *et al*. [17], with lower (10th), median, and upper (90th) percentiles included.

MPR is a very useful addition to concentration values in determining acute poisoning death. In this study, we found the difference in median MPR between poisonings and other causes of death to be diagnostic for all substances with ≥10 cases, excluding clomipramine. Figure 1 shows a comparison with the Swedish study by Reis *et al*. [17] with lower (10th), median, and upper (90th) MPR percentiles included. MPR provides similar or better statistical consistency between data from two different laboratories than obtained for concentrations in our previous article [13]. However, the small number of cases caused differences in the indicators for some substances.

In the following, each antidepressant is briefly discussed in terms of the underlying mechanism of action, fatal toxicity, metabolizing enzymes involved, relation between the established reference plasma concentration range from TDM and the non-poisoning PM concentration from this study, and published PM to AM concentration ratio (PM/AM) where available.

Amitriptyline belongs to the group of TCAs that act primarily by inhibiting the reuptake of serotonin and noradrenaline, but they also have other targets [22]. Like TCAs in general, amitriptyline ranks high in antidepressant fatal toxicity index (FTI) studies [8, 9, 11, 12, 17]. The main active metabolite is nortriptyline, and the major enzyme involved in its formation is CYP2C19. The PM concentrations of amitriptyline and nortriptyline and their sum concentration were significantly higher in all three groups of fatal poisonings than in the other causes of death, and the MPR was significantly lower. In TDM, the monitored active moiety is amitriptyline plus nortriptyline, the therapeutic reference range being 0.08–0.2 mg/L according to Hiemke *et al*. [14]. The median PM concentration of the active moiety in other causes of death (0.61 mg/L) was three times higher than the upper end of the TDM range (0.2 mg/L). The median PM/AM for amitriptyline and nortriptyline in Mantinieks *et al*. [23] was 2.6 and 2.3, respectively, which is consistent with the PM increase found here.

Bupropion is a noradrenaline and dopamine reuptake inhibitor (NDRI). The FTI of bupropion is similarly high to that of TCAs [9, 11]. CYP2B6 catalyzes formation of the major active metabolite hydroxybupropion, which exhibits about 50% of bupropion’s activity [14]. The PM concentrations of bupropion and hydroxybupropion and their sum concentration were significantly higher in all three groups of fatal poisonings than in the other causes of death, and the MPR was significantly lower. In TDM, the therapeutic range of 0.85–1.5 mg/L in Hiemke *et al*. [14] refers to hydroxybupropion only due to instability of bupropion at room temperature. The median PM concentration of hydroxybupropion in other causes of death (0.26 mg/L) was six times lower than the upper end of the TDM range (1.5 mg/L).

*Threo*-hydrobupropion is a stable bupropion metabolite, exhibiting about 20% of bupropion’s activity [14], previously used as a complementary indicator for the extent of exposure to the parent compound [24]. The PM concentrations of bupropion and *threo*-hydrobupropion and their sum concentration were significantly higher in all three groups of fatal poisonings than in the other causes of death, and the MPR was significantly lower. In our study, the median PM concentrations of *threo*-hydrobupropion in all groups of fatal poisonings (4–11 mg/L) and in other causes of death (1.2 mg/L) were fairly similar to those previously reported, 13.5 and 1.5 mg/L, respectively [24].

Citalopram is an SSRI drug, being less toxic in terms of FTI than the TCAs [8–11, 17]. The parent drug is mainly responsible for the pharmacological action, while CYP2C19-catalyzed formation of norcitalopram might contribute only weakly [14]. The PM concentrations of citalopram and norcitalopram and their sum concentration were significantly higher in two groups of fatal poisonings (implicated and principal finding) than in the other causes of death, and the MPR was significantly lower. For single-drug poisonings, the significance was not calculated due to the small number of cases. In TDM, the therapeutic reference range of citalopram is 0.050–0.11 mg/L according to Hiemke *et al*. [14]. The median PM concentration of citalopram in other causes of death (0.37 mg/L) was three times higher than the upper end of the TDM range (0.11 mg/L). The median PM/AM for citalopram in Mantinieks *et al*. [23] was 2.4, which is consistent with the PM increase found here.

Clomipramine is a TCA drug with a high FTI score [8, 9, 12, 17]. The main active metabolite is norclomipramine, and the major enzymes involved in its formation are CYP2C19 and CYP1A2. Clomipramine and norclomipramine differ in their pharmacological profile, the parent being preferential serotonin reuptake inhibitor and the metabolite preferential noradrenaline reuptake inhibitor [14]. The PM concentrations of clomipramine and norclomipramine and their sum concentration were significantly higher in one group of fatal poisonings (implicated) than in the other causes of death, but the MPR was not significantly lower. For the other two groups of poisonings, the significance was not calculated due to the small number of cases. In TDM, the therapeutic reference range of clomipramine plus norclomipramine is 0.23–0.45 mg/L in Hiemke *et al*. [14]. The median PM concentration of clomipramine plus norclomipramine in other causes of death (0.93 mg/L) was two times higher than the upper end of the TDM range (0.45 mg/L).

Doxepin is a TCA drug that has had one of the highest FTI scores of all antidepressants in many studies [8, 9, 11, 12]. The main active metabolite is nordoxepin, and the major enzyme involved in its formation is CYP2C19. The PM concentrations of doxepin and nordoxepin and their sum concentration were significantly higher in two groups of fatal poisonings (implicated and principal finding) than in the other causes of death, but the MPR was not significantly lower. For single-drug poisonings, the significance was not calculated due to the small number of cases. In TDM, the monitored active moiety is doxepin plus nordoxepin, the therapeutic reference range being 0.050–0.15 mg/L as reported by Hiemke *et al*. [14]. The median PM concentration of the active moiety in other causes of death (0.64 mg/L) was four times higher than the upper end of the TDM range (0.15 mg/L).

Fluoxetine is an SSRI drug, being much less toxic in terms of FTI than most antidepressants [8–11, 17]. The main active metabolite is norfluoxetine, and several enzymes are involved in its formation, including CYP2C9 and CYP2C19. The PM concentrations of fluoxetine and norfluoxetine and their sum concentration were significantly higher in one group of fatal poisonings (implicated) than in the other causes of death, and the MPR was significantly lower. For the other two groups of poisonings, the significance was not calculated due to the small number of cases. In TDM, the monitored active moiety is fluoxetine plus norfluoxetine, the therapeutic reference range being 0.12–0.50 mg/L according to Hiemke *et al*. [14]. The median PM concentration of the active moiety in other causes of death (1.0 mg/L) was two times higher than the upper end of the TDM range (0.50 mg/L). The median PM/AM for fluoxetine reported by Mantinieks *et al*. [23] was 1.8, which is consistent with the PM increase found here.

Mianserin is a noradrenergic and specific serotonergic antidepressant (NaSSA), with an FTI score between the TCAs and SSRIs [9, 17]. Mianserin metabolism includes three main pathways, with *N*-demethylation catalyzed mainly by CYP2B6. Normianserin retains antidepressant properties but is less sedating than mianserin [25]. The significance of the PM concentration and MPR results was not calculated due to the small number of cases. In TDM, the therapeutic reference range of mianserin is 0.015–0.070 mg/L ­according to Hiemke *et al*. [14]. The median PM concentration of mianserin in other causes of death (0.17 mg/L) was two times higher than the upper end of the TDM range (0.070 mg/L).

Mirtazapine is a NaSSA type of drug, with an FTI score somewhat higher than the SSRIs [8–11, 17]. One of the main metabolites is normirtazapine, formed via CYP3A4, which has activity similar to that of the parent drug but with lower potency [25]. The PM concentrations of mirtazapine and normirtazapine and their sum concentration were significantly higher in one group of fatal poisonings (implicated) than in the other causes of death, and the MPR was significantly lower. For the other two groups of poisonings, the significance was not calculated due to the small number of cases. In TDM, the therapeutic reference range of mirtazapine according to Hiemke *et al*. [14] is 0.030–0.080 mg/L. The median PM concentration of the active moiety in other causes of death (0.29 mg/L) was four times higher than the upper end of the TDM range (0.080 mg/L). The median PM/AM for mirtazapine reported by Mantinieks *et al*. [23] was 1.5, which is less than the PM increase found here.

Sertraline is an SSRI drug, being much less toxic in terms of FTI than most antidepressants [8–11, 17]. The major metabolite norsertraline, formed via CYP2B6, is unlikely to contribute to the parent drugs’ efficacy or tolerability [14]. The PM concentrations of sertraline and norsertraline and their sum concentration were significantly higher in two groups of fatal poisonings (implicated and principal finding) than in the other causes of death, but the MPR was not significantly lower. For single-drug poisonings, the significance was not calculated due to the small number of cases. In TDM, the therapeutic reference range of sertraline is 0.010–0.15 mg/L in Hiemke *et al*. [14]. The median PM concentration of sertraline in other causes of death (0.25 mg/L) was two times higher than the upper end of the TDM range (0.15 mg/L). The median PM/AM for sertraline according to Mantinieks *et al*. [23] was 2.4, which is consistent with the PM increase found here.

Trimipramine is a TCA drug with one of the highest FTI scores of all antidepressants in many studies [8, 9, 12, 17]. The main active metabolite is nortrimipramine, and the major enzyme involved in its formation is CYP2C19. The significance of the PM concentration and MPR results was not calculated due to the small number of cases. In TDM, the therapeutic reference range of trimipramine is 0.15–0.30 mg/L in Hiemke *et al*. [14]. The median PM concentration of trimipramine in other causes of death (0.88 mg/L) was three times higher than the upper end of the TDM range (0.30 mg/L).

Venlafaxine is an SNRI antidepressant, having an FTI score clearly higher than the SSRI drugs and mirtazapine but lower than the TCAs [8–11, 17]. The predominant active metabolite is *O*-desmethylvenlafaxine, and the major enzyme involved in its formation is CYP2D6. The PM concentrations of venlafaxine and *O*-desmethylvenlafaxine and their sum concentration were significantly higher in all three groups of fatal poisonings than in the other causes of death, and the MPR was significantly lower. In TDM, the monitored active moiety is venlafaxine plus *O*-desmethylvenlafaxine, the therapeutic reference range being 0.10–0.40 mg/L in Hiemke *et al*. [14]. The median PM concentration of the active moiety in other causes of death (1.2 mg/L) was three times higher than the upper end of the TDM range (0.40 mg/L). The median PM/AM for venlafaxine reported by Mantinieks *et al*. [23] was 1.4, which is less than the PM increase found here.

Another venlafaxine metabolite, norvenlafaxine, formed via CYP2C19, does not contribute to pharmacological action. The PM concentrations of venlafaxine and norvenlafaxine and their sum concentration were significantly higher in two groups of fatal poisonings (implicated and principal finding) than in the other causes of death, and the MPR was significantly lower. For single-drug poisonings, the significance was not calculated due to the small number of cases. In TDM, norvenlafaxine is seldom monitored.

The advantages of our study include the relatively large amount of data and the results being obtained from a single accredited laboratory. During the study period, several dozen forensic pathologists, supported by forensic toxicologists, participated in the casework and in the determination of the cause of death and the principal drug finding. The metabolite concentrations presented here are unlikely to have had a significant impact on the determination of the cause of death, thus not causing any bias in the concentration and MPR results. However, as reference concentrations for many parent drugs have been available in the literature for decades, there is always a risk of circular reasoning. Another limitation is that the number of cases is still too small for some antidepressants to calculate statistical significance. Söderberg *et al.* [26] have studied the importance of sample size regarding the robustness of PM reference values. They found that >10 detections are usually needed to differentiate between poisoning cases and controls, and this guideline has been followed in our study.

The measured PM concentrations of antidepressants and metabolites can only be considered as interpretation aids as part of the overall process of determining the cause of death. The pathology of antidepressant poisoning may include, e.g. seizures, serotonin syndrome and cardiac toxicity. Fatal arrhythmia may occur without any evidence of it being found at autopsy or histology. Although MPR values have been found to be diagnostically useful in this study, relying too much on them may result in chronic toxicity being overlooked.

A critical review article on the difficulties associated with the interpretation of PM toxicology has been published recently [27]. The article discusses several complicating factors such as PMR, site-to-site variability, bacterial activity, genetic polymorphisms, tolerance, resuscitation efforts, single- or mixed-drug toxicity, and PM interval. However, the review welcomes the recently published approaches which report PM concentration ranges between fatal and non-fatal cases, and single- and mixed-drug toxicity deaths [13, 18, 28]. We believe that our present study meets the above need by presenting statistically well-organized reference data for more reliable case interpretation.

## Conclusions

Use of reference drug and metabolite concentrations and MPR values from PM blood facilitates interpretation of death cases involving antidepressants. The study confirmed previous findings that the PM concentrations of antidepressants and metabolites are regularly higher than their AM concentrations. The MPR values were consistently lower in fatal poisonings than in other causes of death, but due to the limited number of cases, statistical significance could not always be shown. Our study substantiated the results obtained previously regarding the usefulness of MPR values in PM diagnostics.

## Supplementary Material

bkag012_Supplementary_Data

## Data Availability

The data underlying this article cannot be shared publicly due to the terms of the research agreement.
