# Supplementary material for: Antidepressant metabolite concentrations and metabolite-to-parent drug ratios in postmortem femoral blood
Source: J Anal Toxicol. 2026 Feb 15;50(4):bkag012. doi: 10.1093/jat/bkag012 (PMC13197603; doi:10.1093/jat/bkag012)
Supplement: bkag012_Supplementary_Data [file bkag012_supplementary_data.zip › jat-25-4653-File004.docx]

**Supplementary Table 1.** Drug analysis methods with lower limits of quantification applied to antidepressants and their metabolites in postmortem femoral blood.

| **Substance** | **Method^a^** | **LLOQ (mg/L)^b^** |
| --- | --- | --- |
| amitriptyline | UPLC-PDA-CAD | 0.1 |
| nortriptyline | UPLC-PDA-CAD | 0.1 |
| bupropion | LC-QqQ | 0.01 |
| hydroxybupropion  threo | LC-QqQ | 0.01 |
| *threo*-hydrobupropion | LC-QqQ | 0.01 |
| citalopram | UPLC-PDA-CAD | 0.1 |
| norcitalopram | UPLC-PDA-CAD | 0.05 |
| clomipramine | UPLC-PDA-CAD | 0.1 |
| norclomipramine | UPLC-PDA-CAD | 0.05 |
| doxepin | UPLC-PDA-CAD | 0.05 |
| nordoxepin | UPLC-PDA-CAD | 0.05 |
| fluoxetine | UPLC-PDA-CAD | 0.1 |
| norfluoxetine | UPLC-PDA-CAD | 0.1 |
| mianserin | UPLC-PDA-CAD | 0.1 |
| normianserin | UPLC-PDA-CAD | 0.1 |
| mirtazapine | UPLC-PDA-CAD | 0.1 |
| normirtazapine | UPLC-PDA-CAD | 0.1 |
| sertraline | UPLC-PDA-CAD | 0.05 |
| norsertraline | UPLC-PDA-CAD | 0.05 |
| trimipramine | UPLC-PDA-CAD | 0.1 |
| nortrimipramine | UPLC-PDA-CAD | 0.05 |
| venlafaxine | UPLC-PDA-CAD | 0.1 |
| *O*-desmethylvenlafaxine | UPLC-PDA-CAD | 0.1 |
| norvenlafaxine | UPLC-PDA-CAD | 0.05 |

^a^UPLC, ultra-performance liquid chromatography; PDA, photodiode array detection; CAD, charged aerosol detection; LC, liquid chromatography; QqQ, triple quadrupole mass spectrometry.

^b^LLOQ, lower limit of quantification.
