# Supplementary material for: Antidepressant metabolite concentrations and metabolite-to-parent drug ratios in postmortem femoral blood
Source: J Anal Toxicol. 2026 Feb 15;50(4):bkag012. doi: 10.1093/jat/bkag012 (PMC13197603; doi:10.1093/jat/bkag012)
Supplement: bkag012_Supplementary_Data [file bkag012_supplementary_data.zip › jat-25-4653-File005.docx]

**Supplementary Table 2.** Concentrations (mg/L) of antidepressants and their metabolites, parent drug plus metabolite sum concentrations, and metabolite-to-parent drug concentration ratios, with 10^th^ (p10), median, and 90^th^ (p90) percentiles.

|  |  | Implicated in fatal poisoning | | | | Principal finding in fatal poisoning | | | | Single finding in fatal poisoning | | | | Other cause of death | | | |
| --- | --- | --- | --- | --- | --- | --- | --- | --- | --- | --- | --- | --- | --- | --- | --- | --- | --- |
|  | N total | N | p10 | median | p90 | N | p10 | median | p90 | N | p10 | median | p90 | N | p10 | median | p90 |
| **amitriptyline** | 518 | 171 | 0.41 | 1.7 | 6.1 | 108 | 0.77 | 2.5 | 8.9 | 53 | 1.0 | 3.2 | 10 | 347 | 0.14 | 0.39 | 0.94 |
| nortriptyline |  |  | 0.15 | 0.49 | 1.6 |  | 0.22 | 0.58 | 2.0 |  | 0.25 | 0.75 | 2.6 |  | 0.11 | 0.19 | 0.51 |
| amitriptyline + nortriptyline |  |  | 0.57 | 2.2 | 7.7 |  | 1.1 | 3.4 | 9.9 |  | 1.7 | 4.2 | 12 |  | 0.30 | 0.61 | 1.5 |
| nortriptyline / amitriptyline |  |  | 0.11 | 0.31 | 0.94 |  | 0.084 | 0.27 | 0.79 |  | 0.061 | 0.25 | 0.73 |  | 0.23 | 0.58 | 1.5 |
|  |  |  |  |  |  |  |  |  |  |  |  |  |  |  |  |  |  |
| **bupropion (with hydroxybupropion)** | 212 | 47 | 0.098 | 1.1 | 7.9 | 27 | 0.094 | 2.3 | 11 | 10 | 0.20 | 3.3 | 14 | 165 | 0.030 | 0.12 | 0.53 |
| hydroxybupropion |  |  | 0.082 | 1.8 | 5.6 |  | 0.43 | 2.0 | 7.9 |  | 0.23 | 2.9 | 7.9 |  | 0.060 | 0.26 | 1.4 |
| bupropion + hydroxybupropion |  |  | 0.25 | 3.7 | 13 |  | 1.3 | 4.9 | 14 |  | 0.51 | 6.8 | 21 |  | 0.10 | 0.43 | 2.9 |
| hydroxybupropion / bupropion | 212 | 47 | 0.19 | 0.71 | 8.3 | 27 | 0.17 | 0.59 | 13 | 10 | 0.24 | 0.57 | 5.2 | 165 | 0.45 | 2.0 | 11 |
|  |  |  |  |  |  |  |  |  |  |  |  |  |  |  |  |  |  |
| **bupropion (with *threo*-hydrobupropion)** | 212 | 45 | 0.096 | 1.1 | 8.4 | 26 | 0.091 | 2.3 | 11 | 10 | 0.20 | 3.3 | 14 | 167 | 0.030 | 0.12 | 0.53 |
| *threo*-hydrobupropion |  |  | 0.66 | 4.0 | 14 |  | 0.91 | 4.2 | 18 |  | 0.50 | 11 | 128 |  | 0.22 | 1.2 | 4.9 |
| bupropion + *threo*-hydrobupropion |  |  | 0.93 | 5.3 | 23 |  | 2.4 | 7.7 | 29 |  | 0.78 | 14 | 132 |  | 0.31 | 1.4 | 5.4 |
| *threo*-hydrobupropion / bupropion | 212 | 45 | 0.76 | 3.0 | 27 | 26 | 0.56 | 2.5 | 51 | 10 | 0.99 | 2.8 | 41 | 167 | 2.6 | 9.6 | 37 |
|  |  |  |  |  |  |  |  |  |  |  |  |  |  |  |  |  |  |
| **bupropion + hydroxybupropion + *threo*-hydrobupropion** | 209 | 44 | 1.1 | 8 | 27 | 25 | 2.8 | 8.9 | 35 | 10 | 1.0 | 17 | 140 | 165 | 0.45 | 19 | 7.2 |
|  |  |  |  |  |  |  |  |  |  |  |  |  |  |  |  |  |  |
| **citalopram** | 1316 | 52 | 0.30 | 1.6 | 8.2 | 16 | 1.4 | 3.2 | 16 | 3 | - | 13 | - | 1264 | 0.15 | 0.37 | 0.98 |
| norcitalopram |  |  | 0.12 | 0.31 | 1.1 |  | 0.14 | 0.61 | 1.9 |  | - | 0.56 | - |  | 0.063 | 0.12 | 0.33 |
| citalopram + norcitalopram |  |  | 0.53 | 2.2 | 9.0 |  | 2.0 | 3.8 | 17 |  | - | 14 | - |  | 0.23 | 0.51 | 1.3 |
| norcitalopram / citalopram | 1316 | 52 | 0.049 | 0.20 | 0.69 | 16 | 0.029 | 0.12 | 0.66 | 3 | - | 0.11 | - | 1264 | 0.17 | 0.36 | 0.72 |
|  |  |  |  |  |  |  |  |  |  |  |  |  |  |  |  |  |  |
| **clomipramine** | 33 | 10 | 0.30 | 1.5 | 5.7 | 8 | - | 1.9 | - | 3 | - | 2.5 | - | 23 | 0.15 | 0.40 | 1.8 |
| norclomipramine |  |  | 0.29 | 2.3 | 12 |  | - | 3.2 | - |  | - | 2.9 | - |  | 0.078 | 0.62 | 3.1 |
| clomipramine + norclomipramine |  |  | 0.60 | 3.9 | 18 |  | - | 4.6 | - |  | - | 5.1 | - |  | 0.48 | 0.93 | 4.9 |
| norclomipramine / clomipramine | 33 | 10 | 0.54 | 1.4 | 5.3 | 8 | - | 1.4 | - | 3 | - | 0.91 | - | 23 | 0.17 | 1.8 | 5.0 |
|  |  |  |  |  |  |  |  |  |  |  |  |  |  |  |  |  |  |
| **doxepin** | 74 | 25 | 0.69 | 2.8 | 8.2 | 18 | 0.73 | 2.7 | 8.3 | 6 | - | 4.2 | - | 49 | 0.078 | 0.44 | 12 |
| nordoxepin |  |  | 0.19 | 0.90 | 3.6 |  | 0.18 | 0.78 | 3.7 |  | - | 2.1 | - |  | 0.057 | 0.20 | 0.95 |
| doxepin + nordoxepin |  |  | 1.0 | 4.1 | 11 |  | 0.92 | 4.2 | 12 |  | - | 6.3 | - |  | 0.19 | 0.64 | 1.8 |
| nordoxepin / doxepin | 74 | 25 | 0.14 | 0.29 | 0.95 | 18 | 0.14 | 0.26 | 0.94 | 6 | - | 0.31 | - | 49 | 0.17 | 0.53 | 1.7 |
|  |  |  |  |  |  |  |  |  |  |  |  |  |  |  |  |  |  |
| **fluoxetine** | 117 | 11 | 0.54 | 2.5 | 7.2 | 3 | - | 2.4 | - | 0 | - | - | - | 106 | 0.16 | 0.49 | 1.6 |
| norfluoxetine |  |  | 0.33 | 0.98 | 3.1 |  | - | 0.96 | - |  | - | - | - |  | 0.19 | 0.55 | 1.3 |
| fluoxetine + norfluoxetine |  |  | 0.94 | 3.3 | 9.0 |  | - | 3.3 | - |  | - | - | - |  | 0.42 | 1.0 | 3.0 |
| norfluoxetine / fluoxetine | 117 | 11 | 0.17 | 0.36 | 1.5 | 3 | - | 0.36 | - | 0 | - | - | - | 106 | 0.39 | 1.1 | 2.8 |
|  |  |  |  |  |  |  |  |  |  |  |  |  |  |  |  |  |  |
| **mianserin** | 19 | 3 | - | 0.35 | - | 1 | - | 1.6 | - | 0 | - | - | - | 16 | 0.13 | 0.17 | 0.76 |
| normianserin |  |  | - | 0.15 | - |  | - | 0.15 | - |  | - | - | - |  | 0.11 | 0.18 | 0.55 |
| mianserin + normianserin |  |  | - | 0.87 | - |  | - | 1.8 | - |  | - | - | - |  | 0.26 | 0.39 | 1.3 |
| normianserin / mianserin | 19 | 3 | - | 0.39 | - | 1 | - | 0.09 | - | 0 | - | - | - | 16 | 0.51 | 0.79 | 2.5 |
|  |  |  |  |  |  |  |  |  |  |  |  |  |  |  |  |  |  |
| **mirtazapine** | 107 | 11 | 0.82 | 1.6 | 27 | 4 | - | 1.8 | - | 2 | - | 17 | - | 96 | 0.15 | 0.29 | 0.69 |
| normirtazapine |  |  | 0.11 | 0.47 | 0.94 |  | - | 0.35 | - |  | - | 0.54 | - |  | 0.096 | 0.13 | 0.27 |
| mirtazapine + normirtazapine |  |  | 0.95 | 2.0 | 27 |  | - | 2.2 | - |  | - | 18 | - |  | 0.26 | 0.44 | 0.92 |
| normirtazapine / mirtazapine | 107 | 11 | 0.026 | 0.24 | 0.87 | 4 | - | 0.17 | - | 2 | - | 0.42 | - | 96 | 0.23 | 0.44 | 0.98 |
|  |  |  |  |  |  |  |  |  |  |  |  |  |  |  |  |  |  |
| **sertraline** | 238 | 16 | 0.44 | 1.30 | 14 | 10 | 0.73 | 1.7 | 28 | 8 | - | 1.9 | - | 222 | 0.076 | 0.25 | 0.84 |
| norsertraline |  |  | 0.21 | 1.8 | 16 |  | 0.37 | 2.8 | 25 |  | - | 3.1 | - |  | 0.18 | 0.54 | 2.1 |
| sertraline + norsertraline |  |  | 1 | 3.4 | 35 |  | 2.2 | 3.8 | 41 |  | - | 4.5 | - |  | 0.27 | 0.83 | 2.9 |
| norsertraline / sertraline | 238 | 16 | 0.13 | 1.7 | 4.0 | 10 | 0.13 | 2.2 | 4.3 | 8 | - | 2.7 | - | 222 | 1.2 | 2.3 | 4.6 |
|  |  |  |  |  |  |  |  |  |  |  |  |  |  |  |  |  |  |
| **trimipramine** | 15 | 5 | - | 2.5 | - | 4 | - | 2.6 | - | 2 | - | 2.6 | - | 10 | 0.22 | 0.88 | 2.6 |
| nortrimipramine |  |  | - | 0.78 | - |  | - | 0.65 | - |  | - | 0.72 | - |  | 0.063 | 0.87 | 2.8 |
| trimipramine + nortrimipramine |  |  | - | 4.1 | - |  | - | 3.1 | - |  | - | 3.3 | - |  | 0.39 | 2.1 | 6.4 |
| nortrimipramine / trimipramine | 15 | 5 | - | 0.36 | - | 4 | - | 0.28 | - | 2 | - | 0.28 | - | 10 | 0.081 | 0.52 | 5.4 |
|  |  |  |  |  |  |  |  |  |  |  |  |  |  |  |  |  |  |
| **venlafaxine (with *O*-desmethylvenlafaxine)** | 793 | 117 | 0.41 | 2.6 | 32 | 62 | 1.1 | 6.6 | 50 | 20 | 1.8 | 9.1 | 55 | 676 | 0.16 | 0.55 | 2.1 |
| *O*-desmethylvenlafaxine |  |  | 0.22 | 1.1 | 5.9 |  | 0.26 | 2.2 | 8.3 |  | 0.39 | 3.5 | 13 |  | 0.20 | 0.52 | 1.6 |
| venlafaxine + *O*-desmethylvenlafaxine |  |  | 0.22 | 1.1 | 5.9 |  | 0.26 | 2.2 | 8.3 |  | 0.39 | 3.5 | 13 |  | 1.2 | 1.5 | 2.6 |
| *O*-desmethylvenlafaxine / venlafaxine | 793 | 117 | 0.068 | 0.35 | 1.7 | 62 | 0.066 | 0.26 | 0.83 | 20 | 0.067 | 0.28 | 2.5 | 676 | 0.23 | 1.1 | 2.9 |
|  |  |  |  |  |  |  |  |  |  |  |  |  |  |  |  |  |  |
| **venlafaxine (with norvenlafaxine)** | 204 | 51 | 0.41 | 3.0 | 32 | 33 | 0.9 | 4.3 | 49 | 8 | 1.8 | 12 | 55 | 153 | 0.17 | 1.0 | 2.1 |
| norvenlafaxine |  |  | 0.11 | 0.59 | 3.9 |  | 0.13 | 0.98 | 4.7 |  | - | 1.7 | - |  | 0.070 | 0.22 | 0.94 |
| venlafaxine + norvenlafaxine |  |  | 0.93 | 3.7 | 29 |  | 1.2 | 5.8 | 39 |  | - | 14 | - |  | 0.46 | 1.2 | 3.8 |
| norvenlafaxine / venlafaxine | 204 | 51 | 0.065 | 0.15 | 0.50 | 33 | 0.063 | 0.14 | 0.45 | 8 | - | 0.16 | - | 153 | 0.075 | 0.24 | 0.67 |
|  |  |  |  |  |  |  |  |  |  |  |  |  |  |  |  |  |  |
| **venlafaxine + norvenlafaxine + *O*-desmethylvenlafaxine** | 177 | 45 | 1.1 | 4.3 | 38 | 27 | 1.6 | 6.7 | 44 | 8 | - | 19 | - | 132 | 0.87 | 2.1 | 5.0 |
