# Supplementary material for: Antidepressant metabolite concentrations and metabolite-to-parent drug ratios in postmortem femoral blood
Source: J Anal Toxicol. 2026 Feb 15;50(4):bkag012. doi: 10.1093/jat/bkag012 (PMC13197603; doi:10.1093/jat/bkag012)
Supplement: bkag012_Supplementary_Data [file bkag012_supplementary_data.zip › jat-25-4653-File006.docx]

**Supplementary Table 3**

**CASE EXAMPLES**

The following case examples illustrate the use of reference drug concentrations and metabolite-to-parent drug ratio values for interpretation.

**Case 1:** Death from disease and use of amitriptyline and citalopram

A female, 65 y, was found dead on her couch at home. Two days earlier, she had been seen seemingly in good health, but the next day there was no phone contact. She had previously consumed alcohol heavily but had stopped. She had several medical conditions, including Crohn's disease, hypothyroidism, and the aftermath of fractures in her lower limbs from a distant car accident. She was confused at times, and her family suspected excessive use of medication. PM toxicology revealed in femoral venous blood amitriptyline 0.74 mg/L, nortriptyline 0.86 mg/L (nortriptyline/amitriptyline ratio 1.2), citalopram 0.79 mg/L, norcitalopram 0.50 mg/L (norcitalopram/citalopram ratio 0.63), paracetamol 7.5 mg/L, diazepam 0.11 mg/L, desmethyldiazepam 0.11 mg/L, and pregabalin 5.0 mg/L. Amitriptyline and citalopram concentrations were elevated but not at clearly toxic levels. High MPRs also did not indicate poisoning for either substance. The underlying cause of death was determined to be severe coronary artery disease, the manner of death disease, and a contributing factor stenosis and calcification of the mitral valve.

**Case 2:** Suicidal poisoning involving bupropion

A female, 31 y, was found lifeless on the floor of her home in the morning and attempts to revive her were unsuccessful. The last message from her phone had been sent the previous evening, after which she could not be contacted. Ten years earlier, there had been a suicide attempt with drugs. She had long-term mental health problems and had been in psychiatric care several times, most recently a few weeks before her death, when changes were made to her medication. PM toxicology revealed in femoral venous blood bupropion 3.5 mg/L, hydroxybupropion 1.8 mg/L (hydroxybupropion/bupropion ratio 0.51), *threo*-hydrobupropion 12 mg/L (*threo*-hydrobupropion/bupropion ratio 3.4), olanzapine 0.20 mg/L, norolanzapine 0.097 mg/L, nortriptyline 0.25 mg/L, tramadol 0.48 mg/L, nortramadol 0.20 mg/L, alprazolam 0.014 mg/L, pregabalin 7.0 mg/L, aripiprazole 0.35 mg/L, oxycodone 0.061 mg/L, and lithium 2.5 mg/L. PM biochemistry revealed glucose 5.8 mmol/L in vitreous humor. The high concentrations of bupropion and its metabolites together with the low MPR for both metabolites suggested that bupropion was involved in the poisoning. The underlying cause of death was determined to be bupropion poisoning, the manner of death suicide, and a contributing factor bipolar disorder.

**Case 3:** Death from disease and excessive use of fluoxetine

A female, 65 y, was found dead in her house, after being bedridden with a respiratory infection for over a week. She had multiple medical conditions, including hypertension, diabetes, residual schizophrenia, and paroxysmal atrial fibrillation. PM toxicology revealed in femoral venous blood fluoxetine 3.4 mg/L, norfluoxetine 0.88 mg/L (norfluoxetine/fluoxetine ratio 0.26), metformin 35 mg/L, chlorprothixene 0.37 mg/L, flupentixol 0.013 mg/L, and sitagliptin 0.64 mg/L. PM biochemistry revealed beta-hydroxybutyrate 3.3 mmol/L in vitreous humor and C-reactive protein 20 mg/L in EDTA blood. The high fluoxetine concentration suggested that fluoxetine was involved, while the low MPR suggested an acute dose, however, these findings were not considered in the cause of death by the forensic pathologist. The immediate cause of death was determined to be pneumonia and tissue acidosis, and the interim cause of death respiratory infection. The underlying cause of death was determined to be type 2 diabetes, the manner of death disease, and a contributing factor pyelitis.

**Case 4:** Suicidal poisoning with mirtazapine

A male, 63 y, was found lying on his bedroom bed in his home after neighbors noticed a bad smell and a pile of unread mail. He was suffering from depression and had become a recluse. PM toxicology revealed in femoral venous blood alcohol 0.63‰, mirtazapine 33 mg/L, and normirtazapine 0.23 mg/L (normirtazapine/mirtazapine ratio 0.0070). The very high concentration of mirtazapine with the very low MPR suggested mirtazapine poisoning. The underlying cause of death was determined to be mirtazapine poisoning, the manner of death suicide, and a contributing factor depression.

**Case 5:** Death from disease and high sertraline levels

A female, 71 y, who had been a bedridden patient in a nursing home died in palliative care. She suffered from hypertension, type 2 diabetes, and schizoaffective disorder. She had recently been given broad-spectrum antibiotics for an unspecified infection. PM toxicology revealed in femoral venous blood sertraline 1.9 mg/L, norsertraline 10 mg/L (norsertraline/sertraline ratio 5.3), paracetamol 37 mg/L, olanzapine 0.16 mg/L, diazepam 0.068 mg/L, desmethyldiazepam 0.24 mg/L, oxycodone 0.050 mg/L, noroxycodone 0.023 mg/L, risperidone 0.0043 mg/L, and paliperidol 0.020 mg/L. The high sertraline and norsertraline concentrations suggested poisoning, but the very high MPR spoke against this conclusion. The immediate cause of death was determined to be pneumonia, the underlying cause of death severe coronary artery disease, the manner of death disease, and contributing factors type 2 diabetes, schizoaffective disorder, and high sertraline levels.

**Case 6:** Accidental mixed poisoning involving venlafaxine

A female, 36 y, with known long-lasting drug use was found dead on the couch in her home. PM toxicology in femoral venous blood revealed venlafaxine 3.4 mg/L, *O*-desmethylvenlafaxine 0.22 mg/L (*O*-desmethylvenlafaxine/venlafaxine ratio 0.064), norvenlafaxine 0.33 mg/L (norvenlafaxine/venlafaxine ratio 0.097), methadone 0.52 mg/L, pregabalin 5.8 mg/L, amphetamine 0.40 mg/L, 7-aminoclonazepam 0.086 mg/L, and tetrahydrocannabinol 1.2 µg/L. She was not known to use methadone on a regular basis. The high venlafaxine concentration together with the low MPR for both metabolites suggested that venlafaxine was involved in the poisoning. The underlying cause of death was determined to be venlafaxine-methadone-pregabalin poisoning and the manner of death accident.
